# Comparison of LIAISON QuantiFERON-TB Gold Plus with QuantiFERON-TB Gold Plus

**DOI:** 10.1128/spectrum.04754-22

**Published:** 2023-01-12

**Authors:** Kamran Kadkhoda, Matthew Dee, John Simeone

**Affiliations:** a Immunopathology Laboratory, Robert J. Tomsich Pathology & Laboratory Medicine Institute, Cleveland Clinic, Cleveland, Ohio, USA; b Cleveland Clinic Lerner College of Medicine, Case Western Reserve University, Cleveland, Ohio, USA; Quest Diagnostics Nichols Institute

**Keywords:** LTBI, Quantiferon, IGRA, LIAISON

## LETTER

With the large global burden of latent tuberculosis infection (LTBI), screening for LTBI for various reasons has been widely used, including the low-prevalence settings in which targeted testing is the standard practice (1, 2). Interferon-gamma release assays (IGRAs) are usually preferred over tuberculin skin test given the limitations of the latter (3). Several versions of IGRAs have been introduced to the global market, each time with an improvement including performance characteristics and laboratory operations (4). We previously published an operational feasibility study using the latest version of IGRAs that is commercially available, LIAISON QuantiFERON-TB Gold Plus (DiaSorin Inc.) (LIAISON QFT-Plus) (5). As a sequel to our previous study, we set out to compare LIAISON QFT-Plus with QFT-Plus, including method comparison, intra- and inter-precision and cutoff verification in comparison with the previously used reference method, QuantiFERON-TB Gold Plus (Qiagen) (QFT-Plus). LIAISON QFT-Plus is based on chemiluminescence immunoassay whereas QFT-Plus is based on enzyme immunoassay, and our laboratory did the test on DSX (Dynex technologies) batch analyzers. To this end, 190 randomly-selected, de-identified residual plasma samples were used for the comparisons. The samples were procured based on manufacturer’s instructions and within the established stability requirements in the lab (typically kept at ≤–20°C up to 28 days). Of these stored samples, 97, 23, and 70 had initial negative, indeterminate, and positive results, respectively. For the precision and cutoff verification studies, we compared both TB1 and TB2 result components for which we arbitrarily chose low (0.35 to 1.0 IU/mL) and high (>1.0 IU/mL) levels (one sample from each). For the cutoff verification, a sample was used with initial values within 20% of the positivity cutoff (0.35 IU/mL) and tested 10 times in 1 run.

The results showed acceptable intra- and inter-run precision as well as cutoff verification across the 2 analyzers at the aforementioned levels (Table 1). Overall, the LIAISON QFT-Plus showed acceptable coefficient of variation (CVs) i.e., less than 20% that are generally acceptable for any automated test. Cutoff verification showed CVs for QFT-Plus TB1 and TB2, QFT-Plus LIAISON TB1 and TB2 of 5.2%, 4.1%, 5.2%, 5.4%, respectively, demonstrating robustness around the cutoffs.

**TABLE 1:** Intra- and inter-run precision between the reference (QFT-Plus) versus the test (LIAISON QFT-Plus) methods^*a*^

Precisions	Low TB1 CV (mean)	Low TB2 CV (mean)	High TB1 CV (mean)	High TB2 CV (mean)
Intra-run precision				
DSX	9.0% (0.52)	7.6% (0.49)	0.0% (10.0)	0.0% (10.0)
LIAISON XL	6.4% (0.92)	9.0% (0.93)	0.0% (10.0)	0.0% (10.0)
Inter-run precision				
DSX	15% (0.43)	15.9% (0.43)	0.0% (10.0)	0.0% (10.0)
LIAISON XL	7.3% (0.93)	7.3% (0.94)	0.0% (10.0)	0.0% (10.0)

a For inter-run precision, the sample was run for seven consecutive days. For intra-run accuracy, the same sample was run 10 times in one run. CVs were calculated for each repeat. Analyses were performed by the EP Evaluator software (Data Innovations). Mean values are in IU/mL.

Lastly, the method comparison showed excellent results comparing the 2 kits/platforms (Table 2). In our study, all 20 indeterminate samples that matched between the 2 methods had high Nil (>8 IU/mL) levels. The 3 discordant samples with indeterminate results using QFT-Plus on DSX instrument tested negative on LIAISON QFT-Plus. All 3 samples had had mitogen responses less than 0.5 IU/mL, very low Nil values, and negative TB1 and TB2 results. However, mitogen responses tested all >0.5 IU/mL using LIAISON QFT-Plus. Two of the 3 discordant results were slightly over the acceptable mitogen cutoff (0.5 IU/mL). The third discordant sample with a relatively strong mitogen response suggests that the <0.5 IU/mL result on QFT-Plus was possibly due to an aspiration error, which is not uncommon with the DSX instrument. Suboptimal indeterminate agreement is generally not unexpected; however, indeterminate IGRA results typically pose management dilemmas.

**TABLE 2:** Method comparison between the reference (QFT-Plus) versus the test (LIAISON QFT-Plus)^*a*^

Agreements	QFT-Plus (reference) versus LIAISON QFT-Plus (test)
Negative agreement	100% (97/97)
Indeterminate agreement	86.9% (20/23)
Positive agreement	100% (70/70)
Overall agreement	98.4% (187/190) (95% CI: 95.5 to 99.5%)

a McNemar test for symmetry passed (*P = *0.250 where *P < *0.05 was considered statistically significant) showing no difference between the two methods. Cohen’s kappa was 97.3% (95% CI: 94.2-100.3%) showing a very high agreement between the two methods. The three discordant samples with indeterminate results using QFT-Plus on DSX instrument tested negative on LIAISON QFT-Plus. All three samples had had mitogen responses less than 0.5 IU/mL and very low Nil values and negative TB1 and TB2 results, however, all mitogen responses tested >0.5 IU/mL using the LIAISON QFT-Plus (0.62, 0.99, and 6.97 IU/mL). Analyses were performed by the EP Evaluator software (Data Innovations).

Altogether, the 2 methods were highly comparable. LIAISON QFT-Plus, which is a fully automated, random-access platform is a suitable alternative to semi-automated batch testing (e.g., using DSX) in high-volume laboratories.
